# Let’s walk! Age reattribution and physical activity among older Hispanic/Latino adults: results from the ¡Caminemos! Randomized trial

**DOI:** 10.1186/s12889-018-5850-6

**Published:** 2018-08-03

**Authors:** Lissette M. Piedra, Flavia C. D. Andrade, Rosalba Hernandez, Laura Trejo, Thomas R. Prohaska, Catherine A. Sarkisian

**Affiliations:** 1 School of Social Work, University of Illinois at Urbana-Champaign, 1010 West Nevada St, Urbana, IL 61801 USA; 2 Kinesiology & Community Health, College of Applied Health Sciences, University of Illinois at Urbana-Champaign, Champaign, IL USA; 3 City of Los Angeles Department of Aging, Los Angeles, CA USA; 4 College of Health and Human Services, George Mason University, Fairfax, VA USA; 5 David Geffen School of Medicine at UCLA, Los Angeles, CA USA; 6 VA Greater Los Angeles Geriatric Research Education and Clinical Center, Los Angeles, CA USA

**Keywords:** Hispanic/Latino, Older adults, Physical activity, Intervention, Behavior change, Attribution retraining

## Abstract

**Background:**

Many older Hispanics/Latinos are physically inactive and suffer the harmful health consequences associated with prolonged periods of inactivity. Negative age attributions that equate getting older with “slowing down” reinforce this inactive behavior. We implemented a community-based exercise intervention among insufficiently active older Hispanics/Latinos with a randomized trial of an attribution-retraining program, ¡Caminemos! (Let’s Walk!), and measured the effect of the program on walking behavior.

**Methods:**

Five hundred and seventy-two older Hispanics/Latinos (≥60 years) were enrolled in an exercise program that randomly assigned participants to the exercise class and one of two conditions: (a) treatment (attribution retraining to dispel the notion that physical activity inevitably ceases with age) or (b) control (generic health education). Data were collected at baseline and follow-up (1, 12, and 24 months). Physical activity was determined through pedometer data and the Yale Physical Activity Survey. We also measured the intervention effects on age-expectations, self-efficacy expectations, and outcome expectations for physical activity. Mixed-effects regression models were used to determine intervention effects on prospective measures of physical activity and intrapersonal expectations.

**Results:**

The sample had a mean age of 73 years (SD = 6.8) and was 77% female, and 76% of the sample reported income <$20,000. At baseline, control and treatment groups walked about 3000 steps/day. By 24 months, participants in both arms of the intervention maintained greater than 10,000 mean steps/day, but the difference between the groups was not statistically significant. In analyses adjusted for age, sex, education, income, health status, and acculturation, participants in both trial arms increased their mean numbers of steps at 12 and 24 months, with the treatment group showing a greater number of mean steps compared to the controls at 12 months.

**Conclusions:**

In this group of physically inactive older Hispanics/Latinos, attribution retraining in combination with an exercise class was superior to the exercise class alone with regard to increasing walking behavior. This success was sustained at 12 months (the pre-defined primary study outcome) but not at 24 months. For older Hispanics/Latinos, enrollment in an attribution-retraining exercise program can improve an inactive lifestyle.

**Trial registration:**

clinicaltrials.gov identifier: NCT00183014.

## Background

In the United States, Healthy People 2020 sets forth 10-year national objectives for improving the health of Americans [1, 2]. Featured prominently in this report is a call for dramatically increasing physical activity, levels among older adults, including those with physical and cognitive limitations [1, 2]. Adults 65 years and older spend about 80% of their waking time (10–12 h per day) doing sedentary activities (sitting or reclining) [3–5]. Sitting or reclining requires minimal energy expenditure (1.0–1.5 basal metabolic rate) [6] and poses a significant health risk if daily exposure is prolonged [7–11]. Conversely, physical activity has emerged as an important determinant of functional independence, quality of life, and healthful longevity [12, 13]. Evidence shows that, among older adults, even small amounts of time one can replace sedentary behavior, such as watching TV or sitting in a car, with low, moderate or vigorous physical activity can improve physical function [14, 15]. Regular physical activity can forestall chronic diseases such as obesity [16, 17] and diabetes [18]. Thus, increasing physical activity among older adults is *a public health priority* [19].

Hispanic/Latino older adults, who make up about 8% of the older adult population in the United States and whose numbers are projected to grow to 20% over the next three decades [20–22], are disproportionately sedentary; rates of regular physical activity are reported to be as low as 10% [23] and substantially lower than for non-Hispanic whites [23–26]. Low levels of physical activity serve as an antecedent to multiple chronic diseases for which Hispanic/Latino adults experience elevated risk and associated health disparities. For instance, Hispanic/Latino older adults disproportionately suffer from obesity [27, 28], diabetes [29], and cardiovascular disease [30, 31], contributing to a marked decline in quality of life [32, 33] and higher levels of functional impairments [32].

Pre-existing cultural expectations about older age may contribute to one becoming increasingly inactive with age and may compound the ill effects of other factors, such as low levels of acculturation and education and diminished social support [34, 35]. Some studies have noted that despite acknowledging the benefits of exercise, older Hispanic/Latino adults feel that such exertions are not appropriate, in part because of the perceived risk of injury [36, 37]. Though most people lower their expectations for how healthy they will be as they age, Latinos have especially low age-expectations compared to non-Latino whites and African Americans [38]. These low age-expectations, in particular attributing being sedentary to “old age,” are associated with low rates of physical activity and increased sedentary behavior, which may also contribute to evident health disparities [34, 36, 38–41].

### Beliefs, behavior, and age attribution

To promote physical activity in older adults [42–45] most exercise programs combine multiple elements across theoretical models to inform behavior change [46–48]. Among these theoretical models, social cognitive theory has garnered much empirical support [49, 50]; whether application of attribution theory can be successfully used to change physical activity behavior in seniors has not been examined in a randomized trial to our knowledge [39, 40].

Social cognitive theory [43] posits that behavioral change occurs when goals are set based on: (a) self-efficacy expectations (the belief that one can accomplish a behavior) and (b) outcome expectancy (the belief that engaging in the behavior will deliver positive results). Evidence suggests that raising self-efficacy expectations through setting and achieving goals, behavior modeling, verbal persuasion, and reframing the interpretation of physiological states can encourage physical activity among older adults [49, 51]. In addition, outcome expectancy may be a powerful mediator of physical activity behavior change [52–54]. One study underscored the need for strategies to increase and maintain efficacy within interventions, especially for participants who start out with a lower sense of efficacy [53].

Scholars have long noted that the causal attributions one assigns to outcomes can influence behavioral motivation [39, 40, 55–59]. According to attribution theory, people are more likely to change their behavior when they believe that factors that contribute to outcomes are malleable and within their control [57–59]. According to attribution theory, such designations fall along three dimensions: (a) whether the person identifies an internal or external locus of causality; (b) the stability of the attribution (whether it is perceived as fixed or changeable); and (c) the extent to which the agent perceives that he or she can exert change over the attribution (controllability) [57, 59]. From this perspective, an attribution perceived as fixed and outside the person’s control will demotivate behavioral change [57, 59]. For example, a woman diagnosed as hypertensive and who believes her condition is genetic (it “runs in the family”) invokes a fixed (stable) and uncontrollable attribution. Such an ascription does not inspire behavioral change (to exercise, eat better, or self-monitor one’s blood pressure). Instead, that person might opt merely to take a prescribed medication. However, if the same person reframes her condition as signaling a need for better self-care—an unfixed attribute well within her control—she might be more inclined to initiate health-promoting behavioral changes (diet, exercise, and self-monitoring) to forestall the progression of the condition. Attribution-retraining techniques encourage people to rethink their beliefs so that they come to see outcomes as changeable based on behaviors within their control. We hypothesized that such techniques may be especially promising for older adults who are likely to attribute physical inactivity and health deteriorations to normal “old age.”

**Fig. 1:**
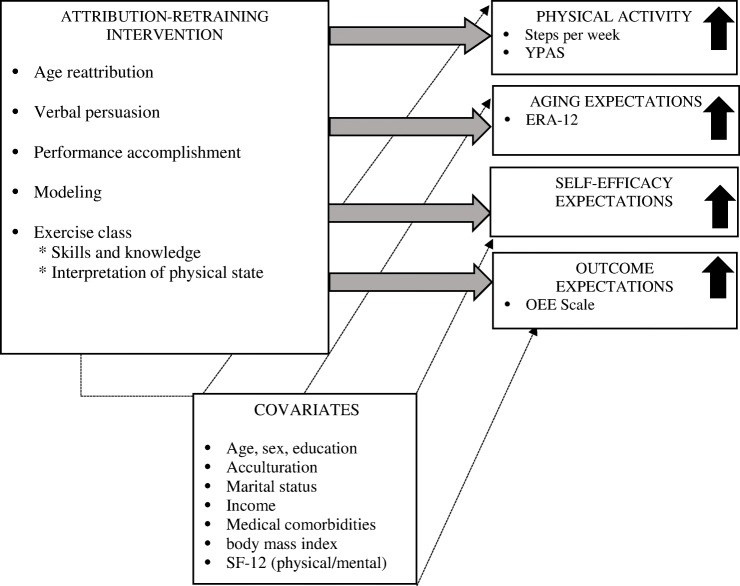
Conceptual framework

Thus, this study combines social cognition theory with attribution theory in an attribution-retraining curriculum to determine whether a random sample of older Hispanic/Latino adults exposed to the curriculum would experience an enhanced response to a modified version of a low-cost exercise program (EnhanceFitness®) when compared to those who received a health education curriculum (see Fig. 1). We hypothesized that exposure to the curriculum would promote higher levels of physical activity compared to those who received generic health education (at 1 month) and that this enhanced performance would be maintained over time (at 12 and 24 months). We also hypothesized that the intervention would have a similar influence on age-expectations, self-efficacy and outcome expectations for exercise among older adults.

## Methods/design

### Trial design

**Fig. 2:**
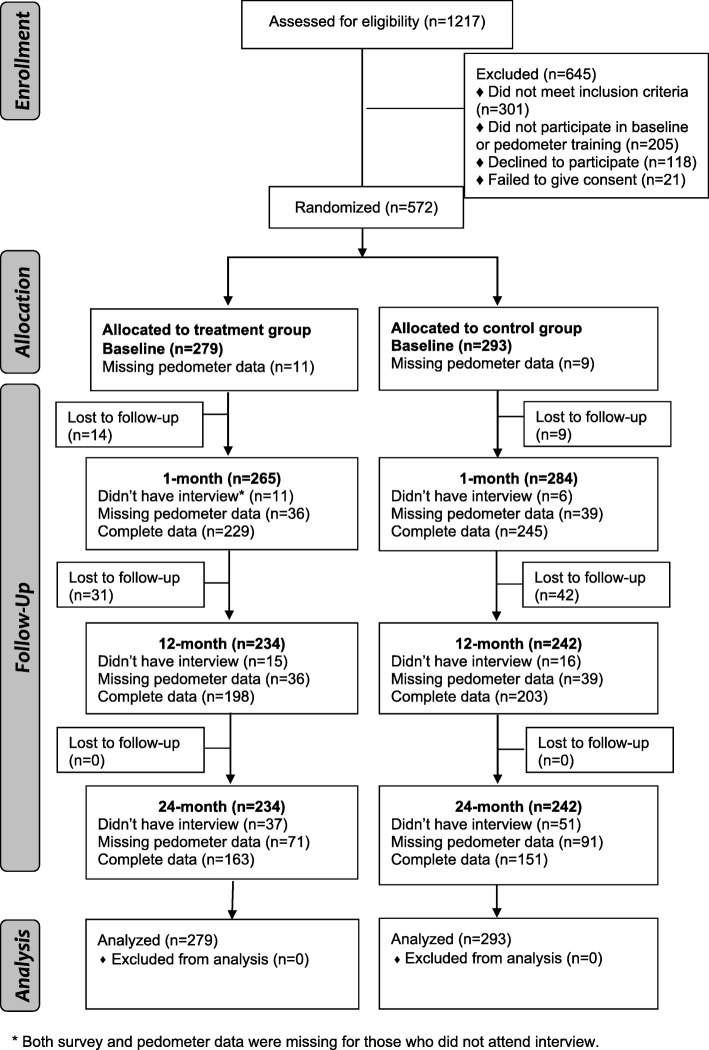
Overview of study design and timeframe

Figure 2 presents a consort diagram of the study design and timeframe. We enrolled 572 Hispanic/Latino older adults in an exercise program and applied a double-blind randomized controlled trial design with two arms: (a) those who received the attribution-retraining curriculum (treatment group) and (b) those who received general health education (control group). Previous studies detail the ¡Caminemos! recruitment and study protocol [39, 60]. The trial was pre-registered at clinicaltrials.gov (identifier NCT00183014). The UCLA Office for Protection of Research Subjects approved the study protocol.

### Participant recruitment and enrollment

Project staff recruited and enrolled participants between August 2005 and August 2007 from 27 community-based senior centers located throughout Los Angeles County that partner with the City and/or County of Los Angeles to provide senior services such as inexpensive mid-day meals, recreational activities, and assistance with social needs such as housing and transportation. Because of the heterogeneity of the Latino population in greater Los Angeles, we decided not to randomize by site; the senior centers differed greatly from each other in terms of site infrastructure characteristics and participant socioeconomic status, activity level, and functional status. In addition, randomization by sites would have been very unattractive to our community partners (those assigned to a control group would not receive the full intervention) and would require a much larger sample size. Thus, randomization occurred at the level of the individual.

We used a two-step protocol that included a face-to-face screening with application of exclusion criteria (Step 1). To be eligible, potential participants had to: (a) be 60 years of age or older, (b) self-identify as Latino, (c) be verbally fluent in English or Spanish, (d) be cognitively intact as determined by a six-item cognitive screener [61], (e) be able to walk (the use of assistive devices such as canes or walkers was not an exclusion criterion); (f) be physically inactive, which was defined as engaging in less than 20 min of exercise at least three times per week; and (g) be available and able to attend weekly exercise and education classes held at the senior center. We set our exclusion criteria of 20 min 3×/week, which is below the recommended levels of 150 min of moderate intensity activity over the week, because our focus was on those seniors most in need of starting a walking program. Potential participants who were eligible based on the Step 1 screening protocol provided the name of a primary care physician who had seen them in the past year; this physician was sent a fax describing the study and asked to send a response fax if the patient had any medical contraindication to participating in a walking program. Potential participants who wanted to enroll but had not seen a physician were offered appointments at local doctors’ offices that accepted sliding scale payments. We excluded spouses or others living with anyone who was also enrolled (due to increased risk of contamination) but allowed spouses/housemates to attend the classes without enrolling (and we did not collect any data on them).

After 1 week had passed without receipt of a fax from the physician indicating a medical contraindication, potential participants were invited to a one-on-one orientation session at the senior center, during which trained staff explained the study and obtained written informed consent to participate. Participants were given a pedometer, trained to use it, and told to return a week later, at which point trained bilingual staff collected baseline data. Of 1217 potential participants screened, 645 (53.0%) were excluded for the following reasons: (a) did not meet the study criteria (*n* = 301), the most common reason being scheduling conflicts due to childcare responsibilities; (b) did not participate in baseline or pedometer training (*n* = 205); (c) declined to participate (*n* = 118); or (d) failed to complete informed consent (*n* = 21) (see Fig. 2).

### Randomization

Randomization occurred once the participants had scheduled their appointment to provide baseline data. We followed an allocation sequence that randomized at the level of the individual (instead of senior centers). Participants were randomized using a random number sequence generator using SAS software (SAS Institute, Cary, NC). Our project staff generated the allocation and a staff-member who was not involved in data collection notified participants of their assignment to the “red” (control) or “orange” (treatment) group after all baseline data collection was completed. Couples were randomized as a unit (only one member of the couple was allowed to enroll in the study, but the other member of the couple could participate in all activities other than data collection). Occasionally a potential participant would be part of our computer randomization but did not complete baseline data collection, leading to a non-equal number of participants for each group.

### Blinding

The labels “red group” and “orange group” were used to “blind” participants to the study design and to the primary hypotheses of the study. All participants and staff involved in data collection were kept blind to which arm of the study participants were allocated. Because it was likely that participants from both arms of the study would know each other and might talk to one another about what they had learned, additional precautions were taken to minimize contamination-biasing results toward the null hypothesis. These safeguards included: (a) keeping all instructors uninformed of the study’s hypotheses, (b) not permitting the exercise instructors to see the attribution-training curriculum or observe a group discussion, and (c) once randomized, keeping participants in both arms of the study separate throughout the intervention and data collection. We held separate exercise classes for orange and red group participants. Participants in both arms of the study were exposed to the same amount of staff contact (8 h over 4 weeks for the first month, 4 h per week for the next 11 months, then once every 2 months for the final 12 months of the study).

### Intervention procedures

After eligible participants were enrolled, provided baseline data, and were randomized into the “red” or “orange” group, they participated for 4 consecutive weeks in a weekly 1-h group discussion session of 8–10 participants led by a bilingual health educator who followed the curriculum for either the attribution retraining (treatment group) or the generic health education (control group). The generic health education group received a series of didactic PowerPoint presentations created by project staff on topics related to senior wellness (e.g. disaster preparedness). In about half the sites (14), the group discussions and the exercise classes were held on the same day; at other sites, they were hosted separately. The scheduling was left was up to the discretion of the sites.

*Exercise program*. In addition to the group discussion session (described in detail below), participants in both arms of the study separately received a 1-h exercise class (weekly for 4 weeks), which targeted muscle strength, endurance, balance, and flexibility. The exercise classes were a modified version of the EnhanceFitness® Program (previously called the Lifetime Fitness Program©) administered by Senior Services (Seattle), designed to be safe for seniors and offering both sitting (chair) and standing options for each exercise [62]. All EnhanceFitness® sessions are designed to be safe for seniors with a wide range of physical capabilities.

#### Intervention (treatment) description

A multidisciplinary team of investigators combined attribution theory with social cognitive theory to develop a standardized curriculum to be delivered by trained health facilitators that underscored the idea that becoming physically inactive should not be an expected part of aging. In one early session, participants were tasked with stating the reasons for being insufficiently active, and then taught to categorize the reasons as either immutable (e.g. being old, having a medical condition) or mutable (e.g. being lazy, not having a partner to exercise with). The trained facilitator taught participants to change their attributions from those that are immutable (in particular, old age) to those that are mutable, and then to problem solve together as a group how to address the mutable reasons for being insufficiently active. Participants established action plans to increase physical activity and made a “promise” to do a specific action before the next meeting (e.g. walk for 15 min every other day). All treatment group participants were encouraged to write down how much walking/exercise they did each day and comment at the beginning of each session on how well they kept their “promise.” They also recorded any obstacles they encountered and the extent to which they were able to overcome the obstacles. Based on data showing that behavioral changes are more likely to be sustained if people are given a chance to ponder how their beliefs have changed [63, 64], at the final core session, facilitators asked each participant to reflect and comment on attitudinal alterations that occurred over the four sessions.

#### Reinforcement schedule

After the 4-week “core intervention” period, all participants in both arms of the study received follow-up (reinforcement) sessions, including both 1-h exercise classes and either the attribution retraining or health education classes. These reinforcement classes met monthly for 11 months after the “core intervention” period and every 2 months for an additional 12 months (total intervention duration = 24 months). Both the treatment and the control groups were exposed to equal amounts of contact time with study staff. During the reinforcement sessions, the treatment group received verbal support for the attribution-retraining concepts they had learned during the 1-month “core” intervention, while the control group received new health education classes.

#### Health educator training and fidelity

Bilingual health educators were recruited from the general community via formal job postings as well as word of mouth. Study personnel trained all potential health educators as group leaders over a 2-day period following a standard curriculum that included general group facilitation techniques as well as step-by-step instructions for each of the four core sessions as well as the reinforcement sessions. Each potential health educator led a “mock” session prior to the start of the study; one potential health educator who did not follow the curriculum correctly was not allowed to lead groups. To measure fidelity to the curriculum, all sessions were audiotaped and reviewed by study personnel to assure that approximately three key points for each session were emphasized; feedback to group leaders was provided as needed.

#### Fotonovela

Halfway through the intervention, we distributed a fotonovela (a photo-dramatized short story) to the treatment group that emphasized the concept that being physically inactive should not be an expected part of normal aging. In the fotonovela, the protagonist, a senior Hispanic/Latina woman, initially states that she is “too old” to exercise but then successfully embarks on walking for regular exercise. She concludes, “Even though I am not young, I realize now that I can control my health and feel better by walking.” We modified the group leader curriculum to include passing out and discussing the fotonovela.

#### Attrition and attendance

We anticipated attrition due to death, illness, or lack of interest. We did not contact those who wished to withdraw formally from the trial. However, participants who stopped attending the program were encouraged to continue by telephone and through in-person meetings at the senior centers. When someone missed a discussion, exercise, and/or data collection, we attempted to reengage the person and obtain data. In such instances, we followed a multifaceted, IRB-approved protocol that guided outreach efforts. Overall, attendance rates were 80% for both arms of the intervention. Each group attended 80% of the group discussions and the exercise classes.

### Data collection/outcome measures

Data collection included pedometer readings, an in-person interview to gather closed-ended survey items, a brief physical exam, and a series of performance measures [65–67]. After baseline, subsequent data collections were conducted at 1, 12, and 24 months, which included completing questionnaires and submitting pedometer recordings. Such data were collected from 474, 401, and 314 participants, respectively. After each data collection session a $25 honorarium was provided.

#### Physical activity

Objective and self-reported measures were used to evaluate physical activity. As an objective measure, the Digiwalker pedometer (Yamax DW-500, New Lifestyles, Inc., Kansas City, MO) was used to calculate the average number of steps taken within a 1-week timeframe. It measures vertical accelerations and, when worn over the hip at waist level, accurately records the number of steps taken within a 3% margin of error compared to direct observation and is substantially better than self-reporting [39, 68–70]. During the weeks when the numbers of steps were recorded, each participant met with project staff at the beginning of the week. Project staff used a standardized script to teach participants how to properly wear and use the pedometer without looking at the step counts; the pedometer was intended as a data collection tool, not as a motivator in itself. Staff reset the pedometer to zero and taped the display window with a label that indicated the meeting date. The participants were instructed not to open their pedometers for the entire week and to return to the senior center at the same time the following week. During that time, the participants were asked to wear the pedometers at all times (7 days), excluding those times when they slept, swam, or bathed. For each participant in the treatment and control groups, the recorded number of steps collected accumulatively over the previous 7 days was downloaded to a computer at a single point during baseline and then at 1, 12. and 24 months. If there were days with no recorded steps, it was assumed that the pedometer was not worn that day, and that information was omitted when calculating the average number of steps over the previous 7 non-zero days with a minimum of 4 days. If there were no 4 days with non-zero steps, the pedometer data were coded as missing.

The Yale Physical Activity Survey (YPAS) [71] was used to measure self-reported perceptions of physical activity in older adults across a wide range of undertakings. The YPAS has two sections that generate three scores. First, participants are asked to assess the total time spent on a list of 25 activities in a typical week during the previous month. Second, participants report the frequency and duration of physical activity in five distinct dimensions: vigorous activity, leisure walking, moving, standing, and sitting. The first section is used to calculate a total time summary index (total time spent doing any of the listed activities) and an energy expenditure summary index (total time spent doing each activity multiplied by a kcal intensity code and summed over all activities). The second section is used to obtain an estimate for the activity dimensions summary score, calculated by multiplying the time spent in each dimension by a weight that ranges from 5 for vigorous activity to 1 for sitting and then adding the weighted totals across all five activity dimensions. The Spanish version of the YPAS has been found to be a valid and reliable measure of activity for older adults [72].

#### Expectations regarding aging (ERA-12)

The ERA-12, a modified version of the ERA-38 [73], measures age-expectations in older adults with demonstrated reliability and validity [74]. Evidence suggests that older adults’ perceptions of aging influence their further health outcomes [75–77]. The survey consists of 12 questions, representing three domains of expectations (four items each): general health, mental health, and cognitive function. A total score for aging expectations is obtained by combining all 12 items [74]. Sub-scale and total scores on the ERA-12 range from 0 to 100, with higher scores indicating higher aging expectations for physical, cognitive, and mental functioning; lower scores indicate lower expectations associated with physical, cognitive, and mental decline [74]. Internal consistency reliability estimates for all scales were reported to exceed 0.74 [74].

#### Lorig self-efficacy for exercise scale (modified)

This instrument consists of four items and uses a Likert scale (1, not at all confident, to 10, totally confident) to measure a person’s confidence in his or her ability to regularly engage in moderately intensive exercise three to four times per week in the future (1-, 2-, 4-, and 8-weeks) without exacerbating preexisting symptoms. This scale has been translated into Spanish version and found to be a valid and reliable measure of self-efficacy for exercise in older adults (α = .92) [78].

#### Outcome expectation for exercise scale (OEE)

The OEE is a nine-item scale that measures the *outcome expectations* for exercise among older adults [79]. Outcome expectations are the beliefs that carrying out a specific behavior—in this case, exercise—will lead to a desirable outcome (e.g. losing weight, reduced glucose levels) or perceived benefits (e.g. feeling energetic or relaxed). Such expectations have been found to be positively associated with exercise behavior [80]. OEE scores range from 1 to 5, with 1 suggestive of low outcome expectations for exercise, and 5 suggestive of strong outcome expectations for exercise. The OEE scale has adequate internal consistency (α = .89) and existing evidence show support for its reliability and validity [79]. The evidence of validity indicates that those who exercised regularly had higher OEE scores than those who did not (*F* = 31.3, *p* < .05) [79]. Moreover, a statistically significant relationship was found between outcome expectations and self-efficacy expectations (*r* = .66) [79].

### Covariates

Sociodemographic measures, health status, and level of acculturation were included as potential confounders. The sociodemographic factors assessed were age, sex, education, marital status, and income. The health status measures included body mass index (BMI), medical comorbidities, and physical and mental quality of life.

#### Sociodemographic measures

Demographic variables included age in years and sex (male [reference group], female). Categorical values were created when capturing the covariates of education (no schooling completed [reference group], ≤eighth grade, or some high school and above), income level (<US$20,000 [reference group], US$20,000 or more, missing income), and marital status (never married [reference group], married, separated/divorced, or widowed).

#### Health status measures

BMI was calculated as weight(kg)/height(m)^2^ and classified as underweight (< 18.5), normal (18.5 to < 25), overweight (25 to < 30), or obese (30 or higher). Because there were relatively few underweight participants (*n* = 4), we combined this group with those of normal weight in the analyses.

Because medical comorbidities affect health outcomes, participants indicated the presence of 16 disorders using a self-administered questionnaire modeled after the Charlson Comorbidity Index [81]. These included any of the following conditions: (a) high blood pressure; (b) heart attack; (c) congestive heart failure; (d) stroke; (e) diabetes; (f) arthritis; (g) hip fracture; (h) fracture of wrist, arm, or spine; (i) lung disease; (j) liver disease; (k) cancer; (l) Parkinson’s disease; (m) coronary artery bypass surgery; (n) Alzheimer’s disease or dementia; (o) depression; and (p) anxiety. Any indicated condition received a score of 1 and was added together with nonexistent conditions, which were marked as 0. The total score was treated as a continuous measure, with scores ranging from 0 to 16.

The 12-Item Short-Form Health Survey (SF-12) provides a generic measure of health status by examining eight health concepts: physical functioning, role limitations due to health problems, bodily pain, general health, vitality, social functioning, role limitations due to emotional problems, and mental health [82]. The instrument generates component summaries for physical and mental health through a principal components analysis. Although SF-12 yields norm-based scores for two broad aspects of health—physical and mental—all items are used to score both summary measures, with a higher score indicative of a better health state [83].

#### Short acculturation scale for Hispanics (SASH)

SASH identifies low and high levels of acculturation, which commonly refers to the process of cultural and psychological change that occurs through intercultural contact [84, 85]. SASH uses a 12-item survey to measure language use, media, and ethnic social relations on a four-point scale [86]. Responses are averaged across the items, and scores range from 1 to 4, with higher scores representing greater acculturation. An overall average score of 2.99 differentiates less acculturated respondents (≤ 2.99) from the more acculturated (> 2.99).

### Data analysis

To assess the success of the randomization, we calculated descriptive statistics for sociodemographic factors, health status, acculturation levels, and pedometer data at baseline for those who were in the treatment and control groups. Results for continuous variables were generated as mean ± SD, and, for the categorical variables; results are given as count and percentage in each category. Comparisons between treatment and control groups were performed with t-test (continuous variables) or chi-square test (categorical variables). All analyses were performed using STATA SE 14.2.

Differences in retention between the treatment and control arms were tested using log-rank test. Two sample t-tests were used to compare the continuous variables over time between the treatment and control arms.

To test the primary study hypothesis concerning the effect of the behavioral intervention on the treatment compared to the control groups over time, we constructed a repeated mixed-effects linear regression [87, 88] to analyze longitudinal changes in the outcome variables as a result of the exercise class and the attribution-retraining component. Repeated mixed-effects regressions allow for an unequal number of observations across individuals, which is an advantage over generalized linear models. Repeated mixed-effects regression models also handle nested data inherent to repeated observations within individuals. All outcome measures were treated as continuous variables. The regression models included treatment group, time in years, and the interaction of group and time, as well as terms for baseline covariates—sociodemographic factors, health status, and acculturation levels. Random effects for the intercept were included to allow individuals to vary in the initial baseline values. In order to facilitate the interpretation of regression results related to the pedometer data, particularly the interaction effects, we examined the linear predictions obtained with the “margins” command and the contrasts involving factor variables and their interactions using the “contrast” command.

## Results

### Baseline characteristics

Nearly 80% of the participants were born outside the United States, and nearly two-thirds of sample participants completed the survey in Spanish. Table 1 displays the baseline distributions of sociodemographic factors, health status and acculturation levels for whole sample and by each arm of the intervention. As a group, the participants ranged in age from 60 to 93, with a mean age of 73.1 (SD = 6.8) and a majority were female (77.1%). More than half the sample had an 8th grade education or less. Only 12.6% had never married. They rest were married (28.8%.), widowed (36.1%) or separated/divorced (22.5%). The majority (75.5%) earned less than $20,000 per year. On average, the participants had 2.6 medical comorbidities and 83.6% had a BMI indicative of being overweight or obese. The median SF-12 scores were 41.5 and 50.3, for physical and mental health, respectively. Levels of acculturation were relatively low with an average mean score of 2.1 points across participants. In the distribution of sociodemographic, health-status and acculturation levels measured at baseline, we found no statistical significant differences between the control and treatment groups.

**Table 1:** Descriptive statistics of individual-level covariates in the ¡Caminemos! study

Variables	All	Group	
		Control	Treatment
	N (%)^a^	N (%)^a^	N (%)*
Mean age (SD)	73.1 (6.8)	73.2 (6.8)	73.1 (6.7)
Sex (*n* = 572)			
Male	131 (22.9)	58 (19.8)	73 (26.2)
Female	441 (77.1)	235 (80.2)	206 (73.8)
Education (n = 572)			
No schooling completed	83 (14.5)	49 (16.7)	34 (12.2)
≤ 8th grade	256 (44.8)	132 (45.1)	124 (44.4)
Some high school or more/other	233 (40.7)	112 (38.2)	121 (43.4)
Marital status (*n* = 570)			
Never married	72 (12.6)	41 (14.0)	31 (11.2)
Married	164 (28.8)	77 (26.4)	87 (31.3)
Separated/divorced	128 (22.5)	61 (20.9)	67 (24.1)
Widowed	206 (36.1)	113 (38.7)	93 (33.5)
Income (n = 572)			
< $20,000	432 (75.5)	221 (75.4)	211 (75.6)
$20,000 and above	90 (15.7)	46 (15.7)	44 (15.8)
Missing	50 (8.4)	26 (8.9)	24 (8.6)
Mean medical comorbidities (SD) (n = 572)	2.6 (1.5)	2.7 (1.8)	2.6 (1.5)
BMI categories (*n* = 569)			
Underweight and normal	93 (16.3)	47 (16.2)	46 (16.5)
Overweight	213 (37.4)	105 (36.1)	108 (38.8)
Obese	263 (46.2)	139 (47.8)	124 (44.6)
Mean SF-12: Norm-based standardization of scale scores (Physical) (SD) (n = 572)	41.5 (9.6)	41.3 (10.3)	41.8 (9.6)
Mean SF-12: Norm-based standardization of scale scores (Mental) (SD) (n = 572)	50.3 (11.3)	49.9 (11.6)	50.7 (11.1)
Mean level of acculturation (SD) (n = 572)	2.1 (1.3)	2.0 (1.3)	2.2 (1.3)

^a^Percent total to 100 across column

### Retention and Fidelity

**Fig. 3:**
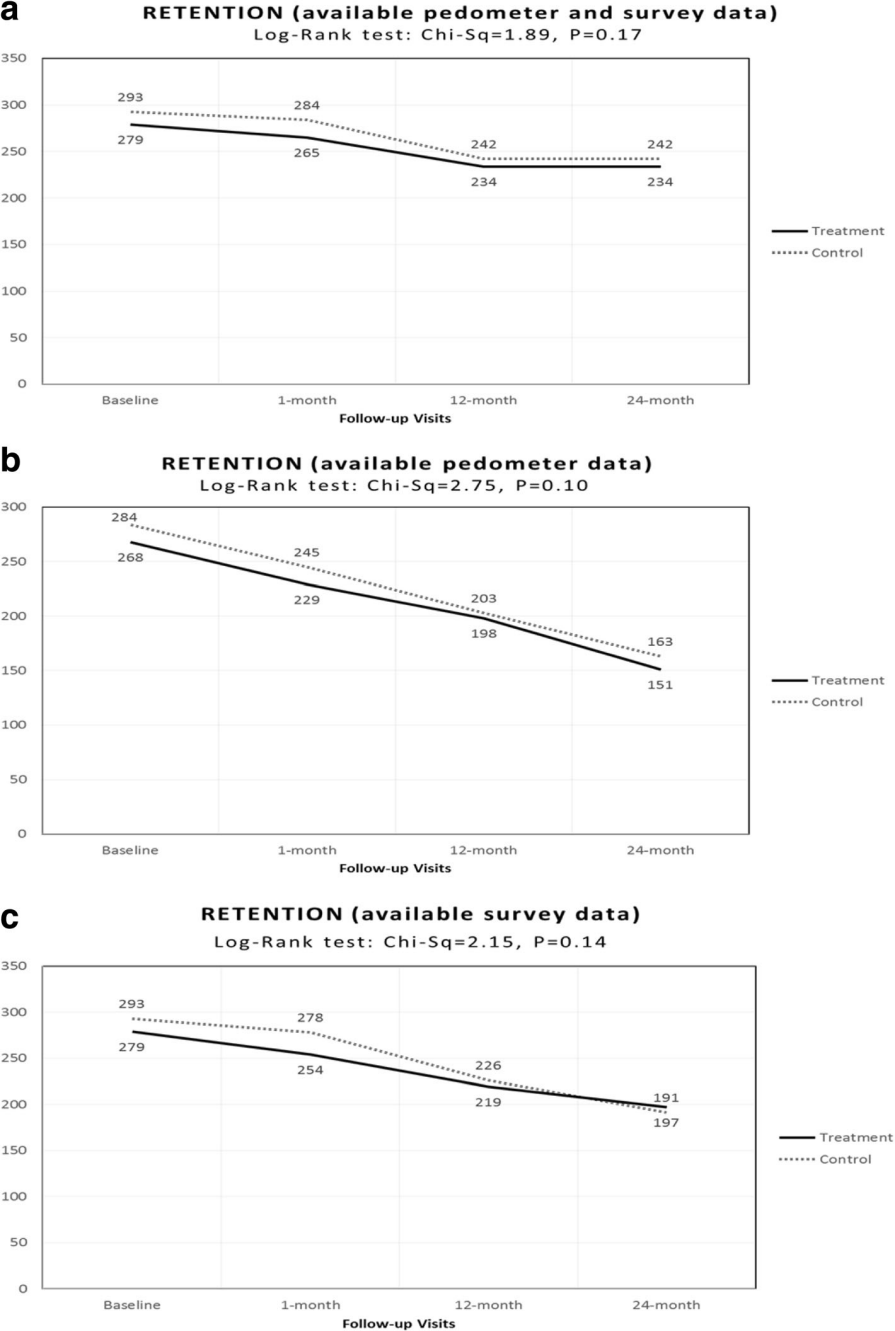
Retention in the ¡Caminemos! Study. **a** RETENTION (available pedometer and survey data). **b** RETENTION (available pedometer data). **c** RETENTION (available survey data)

The results for the study retention showed no statistically significant differences as tested by log rank (x^*2*^ = 1.89, d.f = 1, *p* = 0.17) by group assignment (Fig. 3). The percentage of participants in the treatment group who completed the 2-year study period (84%, *N* = 234 out of 279) was similar to that in the control group (83%, *N* = 242 out of 293). We also found no differences in the retention related to the pedometer data and survey data (Fig. 3).

In addition, research personnel completed fidelity assessments by reviewing audiotapes for 90% of the sessions. Our assessments indicated that the group leaders successfully covered 80% of the key content in the curriculum.

### Changes over time in the outcome measures

Tables 2, 3, 4 and 5 shows the estimates for the treatment and control groups for the outcome measures throughout the study period.

**Table 2:** Descriptive statistics for the outcome measures at baseline and at 1, 12, and 24 months for treatment and control groups: Baseline

Outcomes	Control		Treatment		Difference between two groups		*p*
	Mean	95% CI	Mean	95% CI	Mean	95% CI	
Pedometer	2991.8	(2766.3, 3217.2)	3158.5	(2915.1, 3402.0)	−166.7	(− 497.4, 164.0)	0.3224
YPAS -Total time	10.6	(9.8, 11.5)	11.3	(10.4, 12.2)	−0.7	(−1.9, 0.5)	0.2502
YPAS - Energy expenditure	2328.3	(2116.1, 2540.5)	2533.9	(2281.8, 2786.1)	−205.6	(−533.3, 122.1)	0.2183
YPAS -Activity dimensions	35.1	(32.6, 37.6)	38.3	(35.5, 41.0)	−3.2	(−6.8, 0.5)	0.0898
ERA - Total score	35.3	(33.2, 37.4)	34.8	(32.5, 37.1)	0.5	(−2.6, 3.6)	0.7524
ERA - Physical health scale	30.3	(27.8, 32.7)	29.8	(27.3, 32.3)	0.5	(−3.0, 4.0)	0.7868
ERA - Mental health scale	45.6	(42.6, 48.5)	43.8	(40.7, 46.9)	1.7	(−2.5, 6.0)	0.4242
ERA - Cognitive function scale	30.0	(27.5, 32.6)	30.7	(28.0, 33.4)	−0.7	(−4.4, 3.0)	0.7088
Exercise self-efficacy	8.1	(7.9, 8.3)	8.0	(7.8, 8.3)	0.1	(−0.3, 0.4)	0.7126
Outcome expectation for exercise	4.5	(4.4, 4.5)	4.4	(4.4, 4.5)	0.0	(−0.1, 0.1)	0.3985

**Table 3:** Descriptive statistics for the outcome measures at baseline and at 1, 12, and 24 months for treatment and control groups: 1-month follow-up

Outcomes	Control		Treatment		Difference between two groups		*p*
	Mean	95% CI	Mean	95% CI	Mean	95% CI	
Pedometer	2671.3	(2416.5, 2926.1)	3515.8	(3197.2, 3834.3)	− 844.5	(− 1248.6, − 440.3)	< 0.0001
YPAS -Total time	10.4	(9.6, 11.1)	11.0	(10.2, 11.7)	−0.6	(− 1.7, 0.5)	0.3004
YPAS - Energy expenditure	2279.2	(2096.5, 2461.8)	2419.6	(2219.0, 2620.2)	−140.4	(− 410.4, 129.6)	0.3075
YPAS -Activity dimensions	40.9	(38.3, 43.6)	42.9	(40.0, 45.8)	−2.0	(−5.8, 1.9)	0.3212
ERA - Total score	38.6	(36.3, 41.0)	40.0	(37.5, 42.4)	−1.3	(−4.7, 2.1)	0.4405
ERA - Physical health scale	34.4	(31.8, 37.0)	35.6	(32.9, 38.4)	−1.2	(−5.0, 2.6)	0.5271
ERA - Mental health scale	48.9	(45.6, 52.1)	50.8	(47.5, 54.1)	−2.0	(−6.6, 2.6)	0.3999
ERA - Cognitive function scale	32.6	(29.9, 35.4)	33.4	(30.8, 36.1)	−0.8	(−4.6, 3.0)	0.6769
Exercise self-efficacy	8.3	(8.1, 8.6)	8.4	(8.1, 8.7)	−0.1	(− 0.5, 0.3)	0.6086
Outcome expectation for exercise	4.7	(4.6, 4.7)	4.6	(4.6, 4.7)	0.0	(0.0, 0.1)	0.3786

**Table 4:** Descriptive statistics for the outcome measures at baseline and at 1, 12, and 24 months for treatment and control groups: 12-month follow-up

Outcomes	Control		Treatment		Difference between two groups		*p*
	Mean	95% CI	Mean	95% CI	Mean	95% CI	
Pedometer	8166.5	(7418.9, 8914.1)	9365.4	(8627.9, 10,102.9)	− 1198.9	(− 2246.2, − 151.6)	0.0250
YPAS -Total time	10.6	(9.8, 11.4)	10.7	(9.9, 11.5)	−0.1	(−1.3, 1.0)	0.7951
YPAS - Energy expenditure	2296.8	(2106.2, 2487.4)	2333.8	(2139.4, 2528.2)	−37.1	(−308.6, 234.4)	0.7885
YPAS -Activity dimensions	38.1	(35.3, 40.9)	41.6	(38.6, 44.6)	−3.5	(−7.6, 0.6)	0.0971
ERA - Total score	37.7	(34.6, 40.7)	41.9	(38.9, 44.9)	−4.2	(−8.5, 0.1)	0.0528
ERA - Physical health scale	33.5	(30.2, 36.7)	35.4	(32.0, 38.8)	−2.0	(−6.7, 2.7)	0.4107
ERA - Mental health scale	46.9	(42.9, 51.0)	55.7	(51.9, 59.6)	−8.8	(−14.4, −3.3)	0.0020
ERA - Cognitive function scale	32.6	(29.2, 36.0)	34.5	(31.2, 37.8)	−1.9	(−6.6, 2.8)	0.4176
Exercise self-efficacy	8.4	(8.1, 8.7)	8.3	(8.0, 8.6)	0.1	(−0.3, 0.5)	0.6645
Outcome expectation for exercise	4.7	(4.6, 4.7)	4.7	(4.6, 4.7)	0.0	(−0.1, 0.1)	0.9178

**Table 5:** Descriptive statistics for the outcome measures at baseline and at 1, 12, and 24 months for treatment and control groups: 24-month follow-up

Outcomes	Control		Treatment		Difference between two groups		*p*
	Mean	95% CI	Mean	95% CI	Mean	95% CI	
Pedometer	10,593.9	(9703.6, 11,484.1)	11,604	(10,802.3, 12,405.2)	− 1009.9	(− 2200.9, 181.2)	0.0963
YPAS -Total time	11.6	(10.7, 12.6)	12.3	(11.1, 13.4)	−0.6	(−2.2, 0.9)	0.415
YPAS - Energy expenditure	2504.3	(2253.1, 2755.6)	2724	(2416.6, 3031.5)	−219.7	(− 616.8, 177.4)	0.2774
YPAS -Activity dimensions	42.3	(39.3, 45.2)	44.8	(41.6, 47.9)	−2.5	(−6.8, 1.8)	0.2609
ERA - Total score	39.8	(36.1, 43.6)	42.4	(39.0, 45.8)	−2.6	(−7.6, 2.5)	0.3246
ERA - Physical health scale	35.7	(31.6, 39.7)	38.4	(34.6, 42.3)	−2.8	(−8.4, 2.8)	0.3274
ERA - Mental health scale	52.6	(47.8, 57.3)	55.6	(51.2, 59.9)	−3.0	(−9.4, 3.4)	0.3593
ERA - Cognitive function scale	31.3	(27.1, 35.4)	33.1	(29.4, 36.9)	−1.9	(−7.5, 3.7)	0.5147
Exercise self-efficacy	8.7	(8.3, 9.0)	8.8	(8.5, 9.2)	−0.2	(−0.7, 0.3)	0.429
Outcome expectation for exercise	4.8	(4.7, 4.8)	4.8	(4.7, 4.8)	0.0	(−0.1, 0)	0.4441

#### Pedometer data

**Fig. 4:**
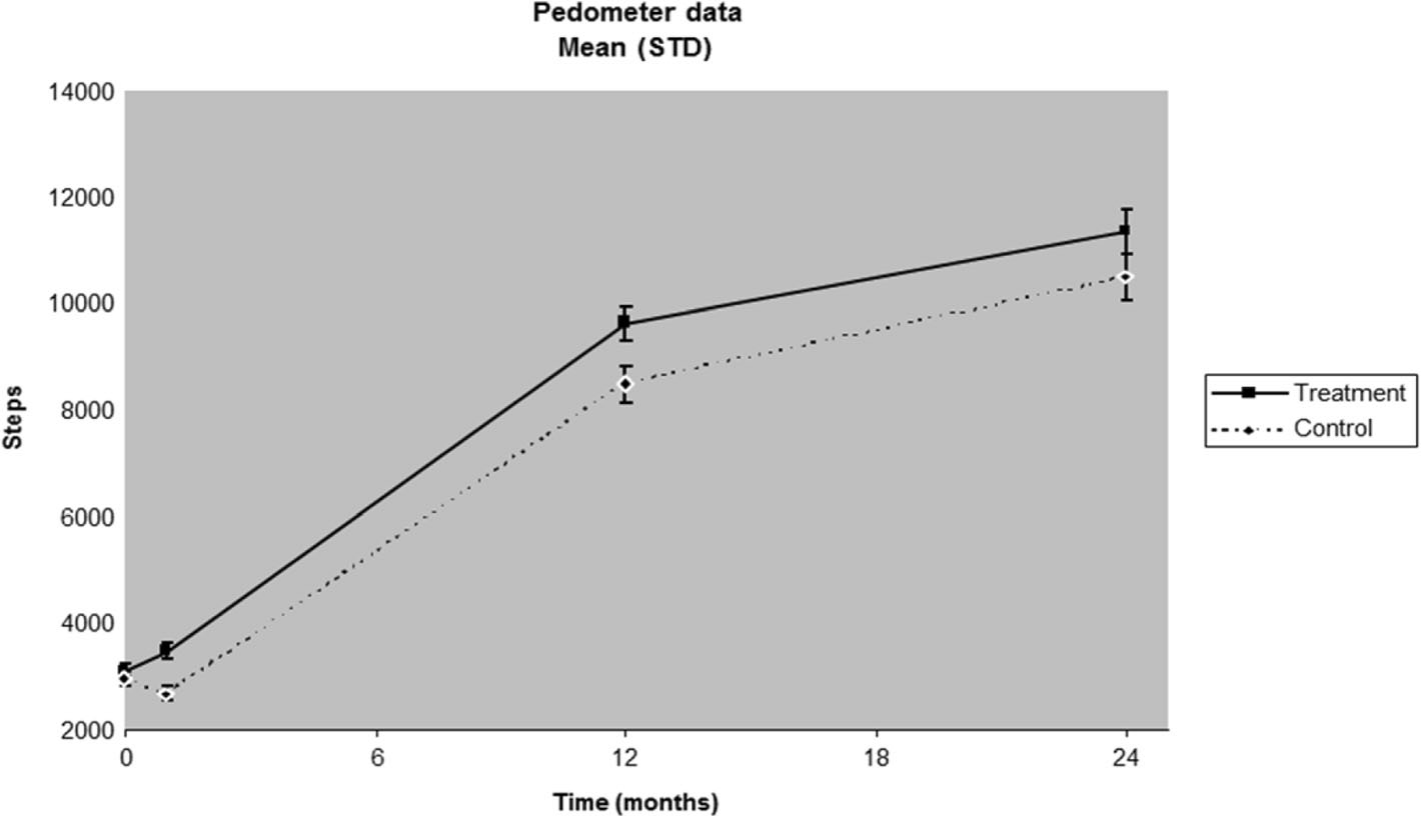
Longitudinal results between the treatment and control groups (mean steps over time)

Figure 4 displays the longitudinal changes in steps between the intervention and control groups. At baseline, the 7-day daily average for participants in both arms of the intervention numbered below 3200 steps, with no statistical difference between the two groups. One month later, the treatment group walked significantly more steps than the control group (3515.8 vs. 2671.3) (*p* < .0001). At 12 months, participants in both arms of the intervention more than doubled their 7-day averages. The treatment group continued to outpace the control group (9365.4 vs. 8166.5) (*p* < .05). By 24 months, participants in both arms of the intervention showed a 7-day average of more than 10,000 steps. Although the treatment group averaged more steps compared to the control group (11,604 vs. 10,593.9 steps), this was not a statistically significant difference (*p* = 0.096).

#### Survey data

With one exception, no differences were found between the treatment and control groups for each of the survey data measures (YPAS, ERA-12, exercise self-efficacy, and outcome expectation for exercise) throughout the study. At the 12-month follow-up, the treatment group displayed greater mental health expectations regarding aging than the control group (55.7 vs. 46.9, *p* < 0.01). Compared to their baseline averages, participants in both arms of the study showed an increase in their average scores for the YPAS, the ERA-12, exercise self-efficacy, and outcome expectations.

#### Adjusted models

Table 6 shows the estimated effects from the mixed-effects linear models (coefficients and 95% CIs) of treatment versus control on outcome measures at baseline and at 1, 12, and 24 months. In analyses adjusted for sociodemographic factors, health status, and acculturation levels, participants in both trial arms displayed greater numbers of steps at the 12-month and 24-month follow-ups when compared to original baseline scores (Tables 6 and 7). Participants in the treatment group showed greater improvement in pedometer steps at 12 months than those assigned to the control group, (6190, 95% CI 5531–6850 and 5099, 95% CI 4450–5749) (Table 7). At 24 months, the difference across trial arms was not significant. Nonetheless, the control group reached 10,564 steps on average (95% CI 6810–8201), and the treatment group averaged 11,458 steps (95% CI 7639–9011) at the 24-month follow-up (Table 7). There were no differences between groups on the YPAS total time or YPAS energy expenditure. Compared to the control, the treatment group had greater increases in ERA total and mental health scores at 12 months.

**Table 6:** Estimated parameters from repeated mixed-effects regressions on selected outcomes

	Pedometer		YPAS -Total time		YPAS - Energy expenditure		YPAS -Activity dimensions	
	Coefficient	95% CI	Coefficient	95% CI	Coefficient	95% CI	Coefficient	95% CI
Fixed effects								
Intervention (ref = control)	80.7	−553.5715.0	0.6	−0.5,1.7	146.3	− 136.9429.5	2.5	−1.1,6.1
Month								
1	− 355	−963.3253.4	−0.2	−1.1,0.6	− 51.5	−278.1175.1	5.8***	2.6,9.0
12	5090.4***	4440.6,5740.1	0	−0.9,0.9	−24.6	− 266.8217.5	3.3	− 0.1,6.7
24	7492.2***	6796.0,8188.3	0.6	−0.3,1.6	93.6	−156.7343.9	6.7***	3.2,10.2
Group x month								
Treatment × 1	661.8	−210.5,1534.1	−0.1	−1.3,1.1	−42.8	− 369.0,283.5	− 1.6	−6.3,3.0
Treatment × 12	1094.3*	168.4,2020.2	−0.6	−1.9,0.7	−173.1	− 518.5172.2	− 0.2	−5.0,4.7
Treatment × 24	831.8	− 145.4,1808.9	0.1	− 1.2,1.4	49.5	−303.5402.4	− 0.9	−5.8,4.1
Age	− 889.3***	− 1319.6,-459.1	−2.5***	− 3.3,-1.6	− 558.4***	−784.1,-332.6	−3.0*	−5.5,-0.5
Female (ref = male)	− 666.1*	− 1180.5,-151.7	2.2***	1.1,3.2	258	−12.7528.7	− 4.4**	−7.5,-1.4
Education (ref = no schooling)								
≤ 8th grade	−158.2	− 776.7460.3	0.8	− 0.4,2.1	213.7	− 109.0,536.3	4.3*	0.7,7.9
Some high school or more/other	−248.3	− 906.9410.3	1.8**	0.4,3.2	423.8*	78.9768.7	4.9*	1.0,8.8
Marital status (ref = never married)								
Married	−56.9	− 754.0,640.3	0.8	−0.7,2.2	271.5	−95.7638.6	2	−2.1,6.2
Separated/divorced	−44	− 756.9668.8	0.2	− 1.3,1.6	36.4	− 337.0,409.8	4.4*	0.2,8.6
Widowed	236.7	− 431.1904.5	0.2	−1.2,1.6	30.2	−319.6380.0	0.1	−3.8,4.0
Income (ref = less than $20,000)								
$20,000 or more	63.5	− 512.9640.0	−0.2	−1.4,1.1	16.8	− 290.4324.0	0.1	−3.3,3.6
Missing	490.4	−241.0,1221.7	−0.9	−2.4,0.7	− 225.3	− 610.6159.9	−2	−6.3,2.3
Medical comorbidities	−180.6*	−328.8,-32.5	− 0.1	− 0.4,0.2	−40.4	− 117.9,37.1	−0.3	−1.2,0.6
Body mass index (ref = underweight/normal)								
Overweight	− 455	− 1054.8144.9	0.2	−1.1,1.5	52.9	− 263.6369.5	− 1.7	−5.2,1.9
Obese	− 605.0*	− 1197.7,-12.4	− 0.1	− 1.3,1.1	−45.3	− 358.5267.9	−1.9	− 5.4,1.6
SF-12 Physical	23.8*	2.3,45.4	0.1***	0.1,0.2	32.6***	21.3,44.0	0.3***	0.2,0.4
SF-12 Mental	7.1	−12.7,26.9	0.1*	0.0,0.1	10.2	−0.2,20.7	0	−0.1,0.1
Acculturation	−28.1	− 195.0,138.9	0	−0.3,0.4	31.7	−56.4119.8	−0.5	−1.5,0.5
Intercept	3642.0***	1742.4,5541.6	1.2	−2.7,5.2	223.2	−771.6,1218.0	24.6***	13.4,35.8
Random effects								
Intercept	7.2***	7.0,7.4	1.4***	1.3,1.5	6.9***	6.8,7.0	2.3***	2.1,2.4
Residual	8.2***	8.1,8.2	1.6***	1.6,1.7	7.2***	7.2,7.3	3.0***	2.9,3.0
Sample size	1780		1955		1955		1953	
	ERA - Total score		ERA - Physical health scale		ERA - Mental health scale		ERA - Cognitive function scale	
	Coefficient	95% CI	Coefficient	95% CI	Coefficient	95% CI	Coefficient	95% CI
Fixed effects								
Intervention (ref = control)	−1.4	−4.6,1.8	−1	−4.7,2.8	−3	−7.2,1.2	−0.2	−4.1,3.6
Month								
1	3.4**	1.0,5.8	4.0**	1.0,7.1	3.5*	0.3,6.6	2.8	−0.1,5.8
12	2.5	−0.0,5.1	3.3	−0.0,6.6	1.6	−1.8,5.0	2.6	−0.6,5.8
24	3.6**	0.9,6.2	4.4*	1.0,7.8	5.7**	2.2,9.2	0.6	−2.6,3.9
Group x month								
Treatment × 1	1.8	−1.6,5.3	1.9	−2.6,6.3	3.6	−0.9,8.2	0	−4.3,4.2
Treatment × 12	4.2*	0.6,7.8	2.3	−2.4,7.0	9.7***	4.9,14.5	0.8	−3.7,5.3
Treatment × 24	3.2	−0.5,6.9	3.6	−1.1,8.4	4.8	−0.1,9.8	1.2	−3.4,5.8
Age	−6.0***	−8.8,-3.3	−6.7***	−9.7,-3.8	−6.1***	−9.6,-2.6	−5.3***	−8.5,-2.2
Female (ref = male)	4.9**	1.6,8.2	2.7	−0.8,6.3	8.5***	4.3,12.7	3.4	−0.4,7.2
Education (ref = no schooling)								
≤ 8th grade	7.9***	4.0,11.8	7.9***	3.6,12.1	10.3***	5.3,15.3	5.4*	1.0,9.9
Some high school or more/other	12.3***	8.1,16.4	10.4***	5.9,14.9	17.6***	12.3,23.0	8.9***	4.1,13.7
Marital status (ref = never married)								
Married	−1.2	−5.6,3.3	−2.3	−7.1,2.5	0.8	−4.9,6.4	−2.3	−7.4,2.9
Separated/divorced	0.5	−4.1,5.0	0.2	−4.7,5.1	1.8	−4.0,7.6	−0.8	−6.0,4.4
Widowed	0.3	−4.0,4.5	−0.4	−5.0,4.2	1.5	−3.9,6.9	−0.4	−5.3,4.5
Income (ref = less than $20,000)								
$20,000 or more	1.7	−2.0,5.5	2.6	−1.4,6.6	2.9	−1.8,7.7	−0.5	−4.8,3.7
Missing	3.1	−1.6,7.7	2.1	−2.9,7.1	5.1	−0.8,11.1	2.1	−3.2,7.5
Medical comorbidities	−0.6	−1.5,0.4	−0.6	−1.6,0.5	− 0.6	−1.8,0.6	−0.6	− 1.7,0.5
Body mass index (ref = underweight/normal)								
Overweight	−1.2	−5.0,2.7	−3.3	−7.4,0.9	−1	− 5.9,3.9	0.8	−3.6,5.2
Obese	0.2	−3.6,4.0	−0.8	−4.8,3.3	0.1	− 4.8,4.9	1.3	−3.0,5.7
SF-12 Physical	0.4***	0.3,0.5	0.4***	0.3,0.6	0.5***	0.3,0.6	0.3***	0.1,0.5
SF-12 Mental	0.4***	0.3,0.6	0.3***	0.1,0.4	0.7***	0.5,0.8	0.3***	0.2,0.5
Acculturation	0.8	−0.2,1.9	−0.1	−1.3,1.0	1.3	−0.1,2.6	1.4*	0.1,2.6
Intercept	−12.2*	−24.2,-0.1	−3.2	−16.2,9.8	−25.6**	−41.0,-10.2	−7.4	− 21.2,6.4
Random effects								
Intercept	2.6***	2.5,2.7	2.6***	2.5,2.7	2.8***	2.7,2.9	2.7***	2.6,2.8
Residual	2.7***	2.6,2.7	2.9***	2.9,3.0	2.9***	2.9,3.0	2.9***	2.8,2.9
Sample size	1956		1956		1956		1955	
			Exercise self-efficacy		Outcome expectation for exercise			
			Coefficient	95% CI	Coefficient		95% CI	
Fixed effects								
Intervention (ref = control)			−0.2	−0.5,0.2	0.0		−0.1,0.1	
Month								
1			0.2	−0.0,0.4	0.2***		0.1,0.3	
12			0.3*	0.0,0.5	0.2***		0.1,0.3	
24			0.4**	0.1,0.6	0.3***		0.2,0.3	
Group x month								
Treatment × 1			0.2	−0.2,0.5	0.0		−0.1,0.1	
Treatment × 12			0.0	−0.3,0.4	0.0		−0.1,0.1	
Treatment × 24			0.4	−0.0,0.7	0.1		−0.0,0.2	
Age			−0.9***	−1.2,-0.6	0.0		−0.1,0.0	
Female (ref = male)			−0.1	−0.4,0.2	0.0		−0.0,0.1	
Education (ref = no schooling)								
≤ 8th grade			0.2	−0.2,0.6	0.0		−0.1,0.1	
Some high school or more/other			0.4	−0.0,0.8	−0.1		− 0.2,0.0	
Marital status (ref = never married)								
Married			0	−0.5,0.4	0.0		−0.1,0.1	
Separated/divorced			0.2	−0.2,0.7	0.0		−0.1,0.1	
Widowed			−0.2	−0.6,0.2	0.0		−0.1,0.1	
Income (ref = less than $20,000)								
$20,000 or more			0.2	−0.1,0.6	0.1		−0.0,0.1	
Missing			0.1	−0.4,0.6	0.1*		0.0,0.2	
Medical comorbidities			−0.1	−0.2,0.0	0.0		−0.0,0.0	
Body mass index (ref = underweight/normal)								
Overweight			−0.3	−0.7,0.1	0.0		−0.1,0.1	
Obese			−0.3	−0.7,0.1	0.0		−0.1,0.1	
SF-12 Physical			0.1***	0.1,0.1	0.0**		0.0,0.0	
SF-12 Mental			0.0*	0.0,0.0	0.0***		0.0,0.0	
Acculturation			0.0	−0.2,0.1	0.0		−0.0,0.0	
Intercept			5.0***	3.8,6.2	4.0***		3.8,4.3	
Random effects								
Intercept			0.3***	0.2,0.3	−1.4***		−1.5,-1.3	
Residual			0.4***	0.3,0.4	−0.9***		−0.9,-0.8	
Sample size			1893		1955			

***p < 0.001; **p < 0.01; *p < 0.05

**Table 7:** Predicted mean number of steps and differences over time in the ¡Caminemos! study

	Predicted mean number of steps	95% CI		Differences to baseline	95% CI		*p*-value
Control							
Baseline	3058	2616	3500				
Month 1	2702	2233	3171	−356	−964	252	0.251
Month 12	8157	7635	8679	5099	4450	5749	< 0.0001
Month 24	10,564	9986	11,142	7506	6810	8201	< 0.0001
Treatment							
Baseline	3133	2681	3585				
Month 1	3440	2956	3924	307	− 318	932	0.336
Month 12	9323	8796	9850	6190	5531	6850	< 0.0001
Month 24	11,458	10,898	12,018	8325	7639	9011	< 0.0001

## Discussion

The ¡Caminemos! community-based randomized trial is the first study to show that attribution retraining works in increasing walking behavior in older Hispanic/Latino adults. Based on both objective and self-reported measures, we found that participants in both arms of the intervention increased their physical activity throughout the study to favorable levels [89] and that those exposed to the attribution retraining experienced greater increases at 12 months than the controls. Such improvements are notable given the low levels of acculturation observed in the sample—a datum further corroborated by the nativity and preferred language of the participants. The fact that older Hispanic/Latino adults, regardless of socioeconomic status, low levels of acculturation, and the presence of preexisting chronic diseases, responded to a low-cost exercise program suggests a promising cost-saving strategy for promoting their general health.

Additional strengths of the study are also notable. It is one of the few studies to include a substantial number of community-dwelling, urban, older Hispanic/Latino adults [90]. The use of objective and subjective measures heightens the validity of the finding that the participants had significantly improved their activity throughout the study. Such a finding suggests that at least among older Hispanic/Latino adults, disparities in physical activity can be reduced by the availability of fitness programs, environments, and social supports that promote physical movement. Such programs go a long way toward undermining the notion that the aging process includes assuming insufficient levels of physical activity.

Elsewhere, we have also noted the beneficial effects of this increased physical activity on cognitive function across both arms of the study over time, regardless of whether the participants’exercise program was supplemented with the age-related attribution retraining [60]. We maintained that such improvements are particularly notable for those on the cusp of clinically significant cognitive impairment; small changes can affect the extent to which one is disabled and for how long one lives with that disability [60]. Similarly, in this study we found that physical activity was also associated with increased mental health over time across both groups, regardless of whether the participants received the attribution intervention. This finding is consist with other studies that have observed the cascading effects of physical activity on wellbeing through its association with better mood, emotional function and mental-health outcomes [91–93]. Moreover, we found that older adults exposed to the age-reattribution intervention showed greater mental health improvements, compared to the controls, by 12-months. In sum, the results from this study are consistent with recent evidence that shows how positive habitual expectations can bolster exercise-induced psychological benefits (impart more enjoyment, improve mood, and reduce anxiety) and facilitate neurophysiological changes by increasing alpha-2 power, as assessed with electroencephalography [94].

Results also indicate that providing opportunities for physical activity are associated with increases in self-efficacy and outcome expectations for exercise. These findings confirm previous studies have shown that physical activity can enhance self-efficacy among older adults, particularly during the intervention [54]. It also has important implications, as older adults with higher levels of physical activity and self-efficacy are more likely to remain physically active [95]. Levels of outcome expectations for exercise also increased during the study, but previous evidence indicate that self-efficacy tends to be more central for the adhering to physical activity than the expectation of outcome [96]. Nonetheless, both indicators increased during the study. However, contrary to our expectations, these increases were similar in both arms of the study and we expected them to be higher among those exposed to the age-reattribution given that it was developed with combination of social cognition theory and attribution theory.

The striking improvement in physical activity across both arms of the intervention warrants further explanation and underscores the limits of the study. Arguably, the benefits of participating in an exercise program muted the benefits of the attribution-retraining program by the 24-month mark. This finding contradicts our expectations and findings from previous studies, which have found that older adults with low age-expectations had lower levels of physical activity [40], and those with more positive views about aging were less likely to reduce their physical activity over time [97]. In fact, older adults with positive stereotypes about aging often have better physical performance, including walking speed [98]. Wolff and colleagues (2014) report the findings of another randomized control trial in which a physical activity intervention was combined with a component of improving positive views on aging [99]. They report that older adults in the experimental group who were exposed to the ‘views-on-aging’ component had more positive views about aging and had increased their physical activity at 10-months [99]. Our results point to similar findings at 12-months in which we report higher expectations regarding aging, better mental health and higher number of steps among older adults in the age-reattribution group, however our study had a longer follow-up and we were able to see that these effects did not last at 24-months.

Regardless of how one feels about the aging process, the act of participating in an exercise program may have superseded any latent feelings of aging with manifest behavior and created a situation in which both the control and treatment groups benefitted from the exercise program. Such a situation is consistent with attribution theory in which the behavior (exercising) alters thinking (one can be active) and this cognitive change reinforces future behavior [49, 51, 52]. Moreover, we speculate that in this context, the health lectures administered to the control group functioned similarly to the reattribution classes that the treatment group received; both the health lectures and the intervention ultimately reinforced the *association* between health and physical exercise. Thus, irrespective of any preconceived notions about age and physical activity, the idea that movement contributes to greater health infused both arms of the intervention and seemed to stick [100]. This dynamic is a limitation of the study design and occurred without reinforcing the content provided to the controls in the health lectures; it would be interesting to know whether repeating the allocutions given to the control group (rather than giving new lectures) would have made a difference between the intervention and the control group. These findings underscore how environmental cues shape thoughts and behavior, and are consistent with other studies that have observed that perceived neighborhood safety and proximity to resources within a community improved levels of physical activity [34, 35, 90, 101]. Accordingly, this study is limited by the absence of a comparison with a true control group—one in which inactive participants received neither an exercise class nor any form of cognitive support. However, such an experiment would raise ethical concerns that our community partners would find untenable.

Throughout this study, we concentrated our efforts on those seniors most in need of starting a walking program; however, such a focus meant that we excluded potential participants who surpassed our activity thresholds (20 min 3xs per week) but who remained well below the recommended levels of physical activity (150 min of moderate intensity activity accumulatively over the week). These semi-active seniors might also have benefited from the intervention; further research should examine whether this intervention can succeed in this more active group. Another useful comparison would have been between groups of elders who already engaged in regular activity at a minimum recommended threshold, usually between 7000 and 10,000 steps [89] and who were randomly assigned to receive the reattribution training or the health lectures. Such a comparison would be useful in understanding the motivating role of the attribution-retraining intervention. In addition, because the data collection ended after 24 months, we have no way of knowing if those who received the attribution retraining might have had an advantage in *maintaining* their physical activity in the long term.

These results must be appraised within the context of some further limitations. Randomization occurred at the individual-level, which increased the risk for contamination. However, this limitation was weighed against three problems that randomizing by site would pose. First, a heterogeneous Latino population in greater Los Angeles led to sites that differed vastly, especially in regards to physical infrastructure and participant socioeconomic status. The presence of such diversity, stratified by location, would have led us to question whether the observed findings were due to differences between sites rather than the intervention itself. Second, randomization by place would have meant that those sites assigned to a control group would not receive the full intervention, a very unattractive prospect to community partners. Finally, the study would have required a much larger sample size. Taken together, we concluded that the complications created by randomization at the site-level outweighed any contamination issues that might occur (but could be minimized) when randomizing at the individual level. Future studies should measure the impact of the intervention in the context of a larger evaluation framework such as RE-AIM. [102–104]

Another limitation refers to the use of pedometer data to assess step counts. Evidence suggests that, compared to accelerometers, pedometers tend to underestimate the number of steps at lower speeds, which can be a problem when studying frail older adults [105]. In particular, pedometers have problems estimating step counts when individuals have variable gait patterns, which tend to be more common among nursing home residents than among community-dwellers [106]. Therefore, the use of pedometers has shown to be appropriate among community-dweller older adults, although it may be associated with an underestimation in the number of steps [106]. Our estimates based on the pedometer data should be taken as conservative estimates as they may underestimate the real number of steps (by 7–25%) [106] in both arms of the study. Although multiple measures were used, the self-report instruments were administered repeatedly and therefore were vulnerable to repeat testing bias. The responses might have been affected by memory or by a wish to present oneself in a socially acceptable manner, which may include reporting what is perceived as desirable. These factors can lead people to overestimate or underestimate data and bias the results. In addition, we did not include any clinically objective biomarkers that would correspond with enhanced physical fitness. Nonetheless, well-validated instruments were used that have been known to yield robust findings with an understudied population. Moreover, the use of the pedometer data and a consistent pattern of improvement for all measures across the intervention arms indicated that the repeat testing bias exerted a minimal effect. Data on chronic conditions were only available at baseline; therefore, it was not possible to evaluate how they might have influenced the outcomes over the duration of the study. Finally, women composed most of our sample, so one should be cautious when generalizing our findings.

## Conclusion

Overall, although participants in both arms of the intervention benefitted from the exercise intervention, those exposed to attribution retraining outperformed those who received only the exercise program by 12 months. As the Latino population is expected to grow exponentially over the coming decades, increasing physical activity may be a powerful cost-saving strategy for improving the health of older Hispanic/Latino adults, regardless of socioeconomic status, low levels of acculturation, and the presence of preexisting chronic diseases.

## Abbreviations

BMI: Body mass index
ERA-12: Expectations Regarding Aging
IRB: Institutional Review Board
OEE: Outcome Expectation for Exercise Scale
SASH: Short Acculturation Scale for Hispanics
SF-12: 12-Item Short-Form Health Survey
YPAS: Yale Physical Activity Survey
